# Seroprevalence of four endemic human coronaviruses and, reactivity and neutralization capability against SARS-CoV-2 among children in the Philippines

**DOI:** 10.1038/s41598-023-29072-3

**Published:** 2023-02-09

**Authors:** Yusuke Sayama, Michiko Okamoto, Mayuko Saito, Mariko Saito-Obata, Raita Tamaki, Christine Dahlia Joboco, Socorro Lupisan, Hitoshi Oshitani

**Affiliations:** 1 Department of Virology, Tohoku University Graduate School of Medicine, 2-1 Seiryo-Machi, Aoba-Ku, Sendai, Miyagi 980-8575 Japan; 2 Institute of Tropical Medicine, Nagasaki University, Nagasaki, Japan; 3 Biliran Provincial Hospital, Biliran, Philippines; 4 Research Institute for Tropical Medicine, Metro Manila, Philippines

**Keywords:** Antibodies, Viral infection, Paediatrics

## Abstract

Four endemic human coronaviruses (HCoV), HCoV-229E, HCoV-NL63, HCoV-HKU1, and HCoV-OC43, are closely related to SARS-CoV-2. These coronaviruses are known to infect humans living in temperate areas, including children under 5 years old; however, the seroprevalence of four HCoVs among children in tropical areas, including the Philippines, remains unclear. This study aimed to assess the prevalence of antibodies against four HCoVs and to determine the reactivity and neutralization of these antibodies against SARS-CoV-2 among children in the Philippines. A total of 315 serum samples collected from 2015 to 2018, before the emergence of SARS-CoV-2, in Biliran island, Philippines, were tested for the presence of antibodies against four HCoVs and SARS-CoV-2 using recombinant spike ectodomain proteins by IgG-enzyme-linked immunosorbent assay (ELISA). Reactivity to and neutralization of SARS-CoV-2 were also investigated. The seroprevalence of the four HCoVs was 63.8% for HCoV-229E, 71.4% for HCoV-NL63, 76.5% for HCoV-HKU1, and 83.5% for HCoV-OC43 by ELISA. Age group analysis indicated that seropositivity to all HCoVs reached 80% by 2–3 years of age. While 69/315 (21.9%) of the samples showed reactive to SARS-CoV-2, almost no neutralization against SARS-CoV-2 was detected using neutralization assay. Reactivity of antibodies against SARS-CoV-2 spike protein obtained by ELISA may not correlate with neutralization capability.

## Introduction

Coronaviruses (CoV) belong to the subfamily *Coronavirinae* in the family *Coronaviridae* (order, Nidovirales) and are composed of an envelope and a single-stranded, positive-sense RNA molecule of ~ 30,000 nucleotides^1,2^. Members of this subfamily are further classified into alpha-, beta-, gamma-, and delta-coronaviruses. Among CoVs that infect humans, human (H)CoV-229E and HCoV-NL63 belong to the alpha-coronaviruses, whereas HCoV-HKU1, HCoV-OC43, severe acute respiratory syndrome coronavirus (SARS)-CoV-1, Middle East respiratory syndrome (MERS)-CoV, and SARS-CoV-2 are beta-coronaviruses^3–9^. The spike proteins of the CoVs are constitutive of the virions and influence the biological characteristics of the virus, including their antigenicity and pathogenicity^10^.

Four endemic HCoVs, HCoV-229E, HCoV-NL63, HCoV-HKU1, and HCoV-OC43, usually cause mild to moderate upper-respiratory tract illness, like the common cold. In some cases, HCoV infection can cause severe illness, such as croup, bronchiolitis, and pneumonia in the elder, children, and immunocompromised individuals^11,12^. In contrast, SARS-CoV-1 and MERS-CoV are highly pathogenic beta-coronaviruses that often cause very severe illness with high mortality rate among infected individuals^9,13^. The beta-coronavirus, SARS-CoV-2, is the causative agent of the coronavirus disease 2019 (COVID-19)^8^. According to the World Health Organization, this pandemic resulted in more than 600 million worldwide cases of SARS-CoV-2 infection by October 2022 (https://covid19.who.int/).

To date, the seroepidemiology of the four endemic HCoVs among children has been reported from the temperate areas including China, the Netherlands, and the United States^14–16^. However, the seroprevalence of these viruses in tropical areas such as Southeast Asia remains unknown. At the beginning of the COVID-19 pandemic, most SARS-CoV-2-infected children experienced mild or asymptomatic infections, while older adults were disproportionately affected by COVID-19 in terms of severity^17,18^. The COVID-19 epidemiologic peculiarities among children have generated much interest and have raised questions about the possible protection conferred by antibodies elicited by previous infections with other HCoVs. Recently, it was reported that some antibodies produced during HCoV infection could cross-react with SARS-CoV-2^19,20^, but the neutralizing efficacy of these reactive antibodies has been poorly documented, especially in children.

To address these questions, we investigated the seroprevalence of four HCoVs and, reactivity and neutralization capability against SARS-CoV-2 in 315 serum samples collected from children less than 5 years old who presented with severe respiratory illness before the COVID-19 pandemic in the Philippines. The reactivity of the samples to SARS-CoV-2 was measured by a neutralization assay using live virus. Our study clarifies the seroprevalence of four HCoVs in a tropical area, namely, the Philippines, as well as their SARS-CoV-2 reactivity and neutralization capability.

## Results

We investigated a total of 315 serum samples from children in the Philippines from 2015–2018 using four HCoVs and SARS-CoV-2 spike proteins by IgG-enzyme-linked immunosorbent assay (ELISA) (Supplementary Table S1 and Supplementary Fig. S1). This analysis revealed 201 (63.8%) samples that were positive for HCoV-229E, 225 (71.4%) samples that were positive for HCoV-NL63, 241 (76.5%) positive samples for HCoV-HKU1, and 263 (83.5%) positive samples for HCoV-OC43, while 69 (21.9%) samples were positive for SARS-CoV-2. Twenty-five (7.9%) samples were negative for all five antigens.

**Figure 1:**
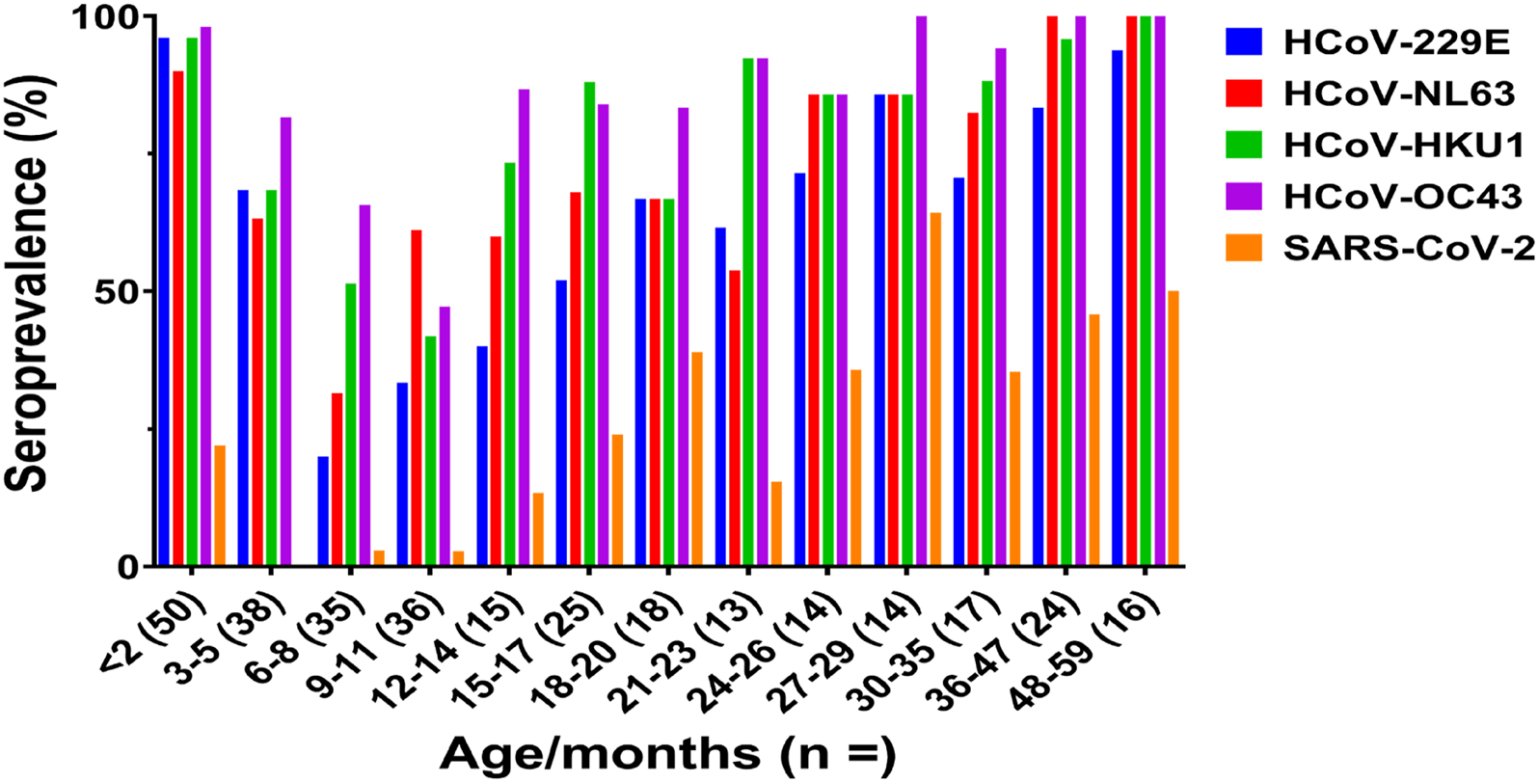
Seroprevalence of four endemic HCoVs and SARS-CoV-2 among different age groups of children who were collected before the COVID-19 pandemic (2015–2018) in the Philippines. The bar chart represents the percentage of serum samples that were positive for HCoV-229E (blue), HCoV-NL63 (red), HCoV-HKU1 (green), HCoV-OC43 (purple), and SARS-CoV-2 (orange) in each age group. The number of samples per age group is indicated in brackets.

The distribution of anti-spike IgG to the four HCoVs among the age groups is shown in Fig. 1 and Supplementary Table S2. We could detect anti-HCoVs antibodies in most of the samples from the 0–2 months old group (96% for HCoV-229E, 90% for HCoV-NL63, and 98% for both HCoV-HKU1 and HCoV-NL63). The seropositivity rate for the four HCoVs decreased for the samples after 3-month-old children and lowest in those from 6–8 months or 9–11 months old. At age 12 to 17 months, 80% samples were positive for beta-coronaviruses (HCoV-HKU1 and HCoV-OC43), while this level of positivity was reached at 24 to 29 months old for alpha-coronaviruses (HCoV-229E and HCoV-NL63), suggesting that the plateau of seropositivity rate for beta-coronaviruses was reached earlier than for alpha-coronaviruses. Although the serum samples analyzed were collected before 2019 (pre-pandemic), we detected SARS-CoV-2 antibodies by ELISA. The seroprevalence with SARS-CoV-2 increased after 12 months old. Furthermore, 20 of the 25 (80%) samples that were negative against the four HCoVs and SARS-CoV-2 were from children aged from 3 to 9 months old.

**Figure 2:**
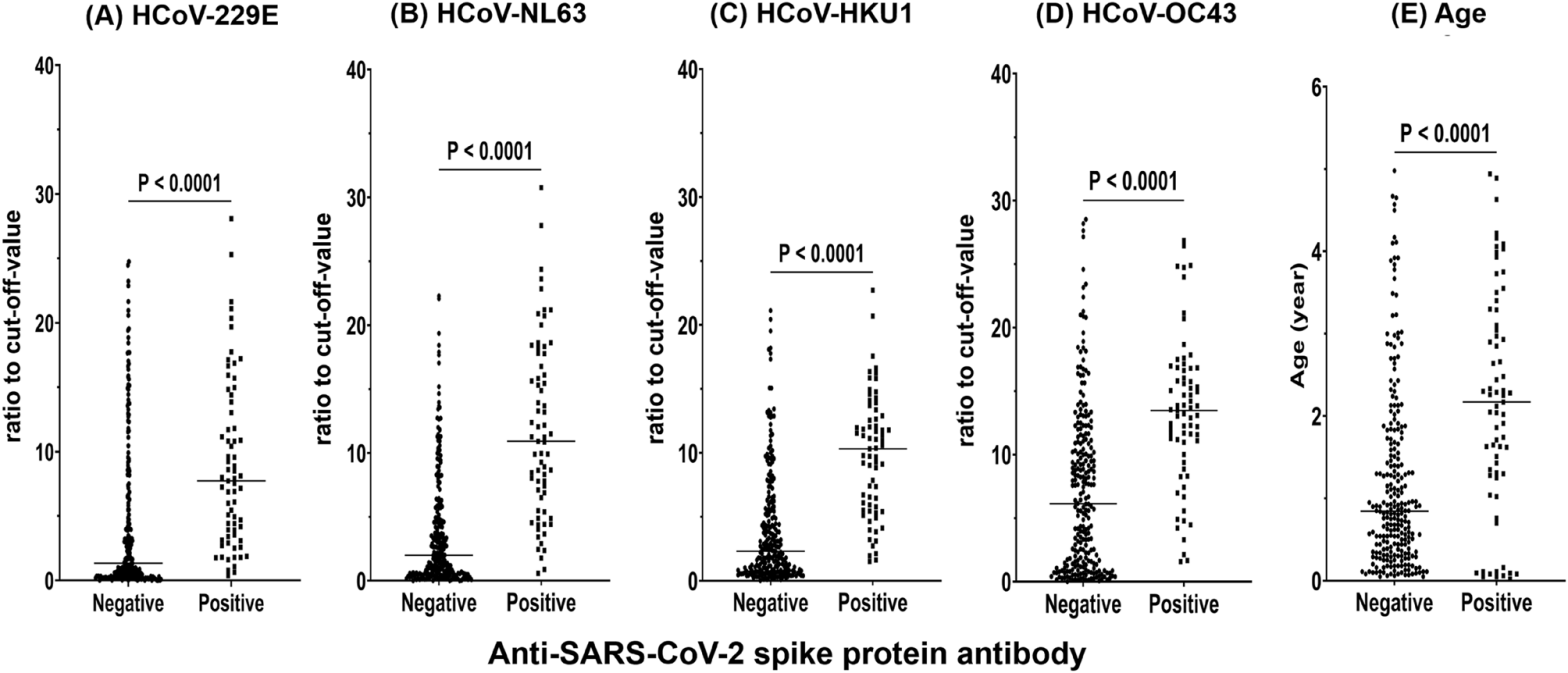
Anti-SARS-CoV-2 antibodies, assessed by ELISA, according to seroreactive to four HCoVs and age in children who were collected before the COVID-19 pandemic (2015–2018). Seroreactive of the sera to (**a**) HCoV-229E, (**b**) HCoV-NL63, (**c**) HCoV-HKU1, and (**d**) HCoV-OC43, assessed by ELISA against the respective spike proteins of the different viruses. (**e**) Anti-SARS-CoV-2 antibodies according to age (negative: n = 246; positive: n = 69). The Y axis represents the ratio to cut-off-value of each antigen, or age, compared with Mann–Whitney U tests. Horizontal lines indicate mean values.

Next, we investigated the reactivity to SARS-CoV-2. We analyzed antibody levels (ratio to cut-off-value) to directly compare the levels of HCoV antibodies in a subset of samples from individuals who either possessed (n = 69) or did not possess (n = 246) reactive SARS-CoV-2 antibodies (Fig. 2a–d). The ratios of cut-off-value for the four HCoVs were significantly higher in individuals with SARS-CoV-2-reactive antibodies than in individuals who did not have SARS-CoV-2-reactive antibodies (*P* < 0.0001). In addition, individuals with SARS-CoV-2-reactive antibodies were older than those ones without SARS-CoV-2-reactive antibodies (*P* < 0.0001) (Fig. 2e).

**Figure 3:**
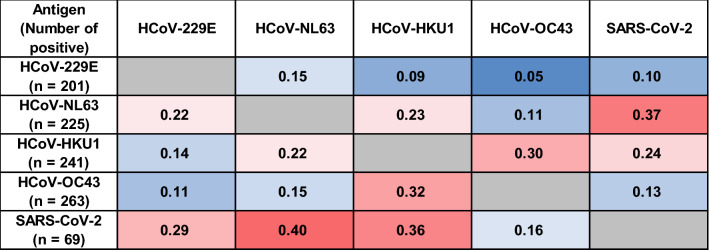
Correlation analysis of the cut-off-value ratio to the four HCoVs and to SARS-CoV-2 in children who were collected before the COVID-19 pandemic (2015–2018). Positive correlations are symbolized by graded shades of red, and negative correlations are by graded shades of blue. The coefficient of determination (R^2^) for each pair of viruses are indicated.

We calculated the Pearson coefficient of determination between the cut-off-value ratios of the positive samples for each antigen against all other antigens (Fig. 3). The coefficient of determination was higher for viruses within the same genus, i.e., between alpha-coronaviruses (HCoV-229E versus HCoV-NL63) than for those within different genera (HCoV-HKU1 and HCoV-OC43). In contrast, the coefficient of determination between HCoV-NL63 positivity and that to other viruses were higher for HCoV-HKU1 (r^2^ = 0.23) and, especially, SARS-CoV-2 (r^2^ = 0.37), which are beta-coronaviruses, compared with the alpha-coronavirus, HCoV-229E. Reciprocally, for SARS-CoV-2, the highest coefficient of determination was scored for HCoV-NL63 (r^2^ = 0.4), while the lowest coefficient of determination was scored for HCoV-OC43 (r^2^ = 0.16).

**Figure 4:**
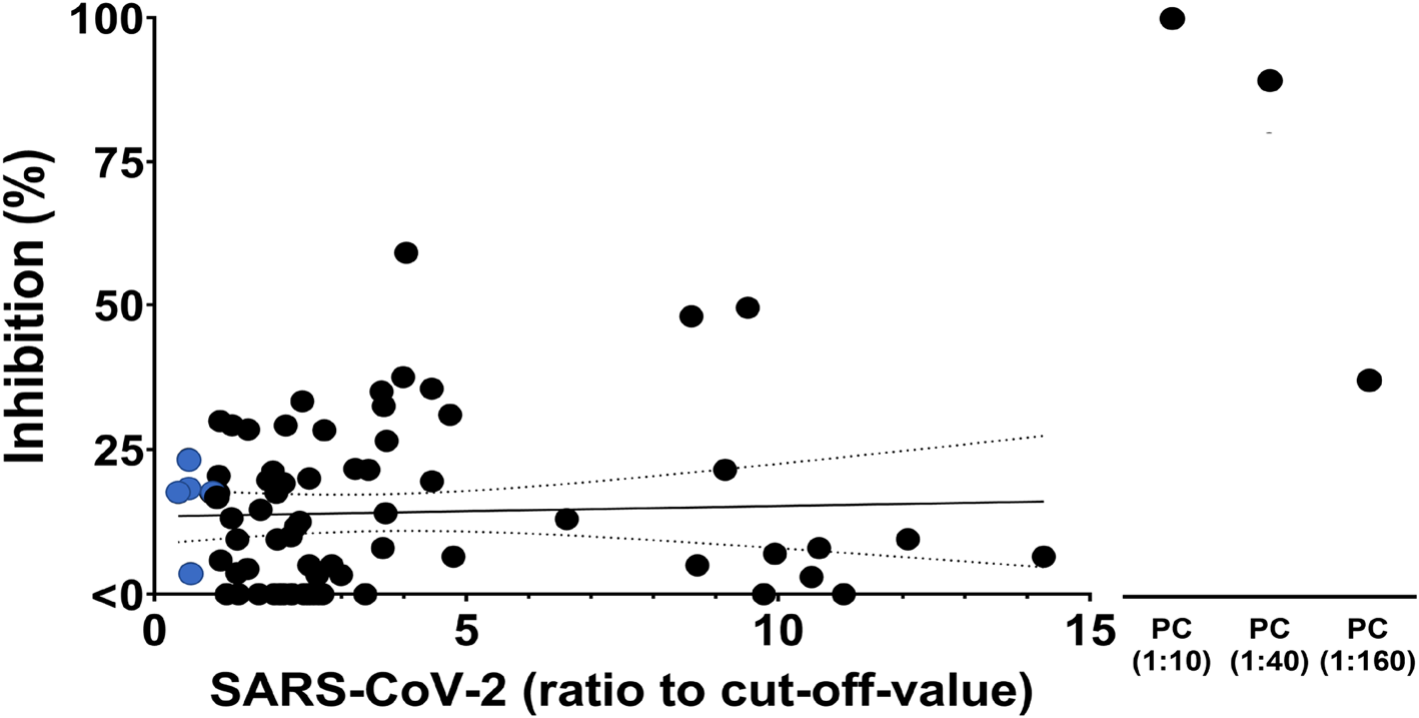
Assessment by PRNT of the neutralizing effect of the SARS-CoV-2 reactive sera. Percentage of inhibition of SARS-CoV-2 infection by reactive sera determined by PRNT in comparison with vehicle control. For each serum, the dot plot shows the correlation between the anti-SARS-CoV-2 reactivity, assessed by ELISA, and the neutralization, assessed by PRNT. A sample from a SARS-CoV-2-infected individual was used as a positive control (PC). SARS-CoV-2 non-reactive samples (negative) are represented with blue circles (n = 5), SARS-CoV-2 reactive samples (positive) are represented with black circles (n = 69). Data are compared by Spearman’s rank correlation coefficient.

Finally, we tested whether the SARS-CoV-2 reactive antibodies were neutralizing antibodies. We tested the 69 SARS-CoV-2 reactive samples using two neutralization assays. In the tissue culture infectious dose (TCID)_50_-based neutralization assay, virus neutralization can be measured by means of reduced visual CPE of the virus on culture cells. In all 69 reactive samples, we could observe CPE using the TCID_50_-based neutralization assay, suggesting that these reactive antibodies are not able to neutralize SARS-CoV-2. Next, we confirmed these results by testing the SARS-CoV-2 reactive samples and five SARS-CoV-2 non-reactive samples using the plaque reduction neutralization test (PRNT). In the PRNT, 68 out of 69 (98.6%) reactive samples did not achieve 50% inhibition. Only one sample (1.4%) showed 59% inhibition of SARS-CoV-2 (Fig. 4). All five non-reactive control samples showed low inhibition (3.6–23.4%). We found no correlation between the cut-off-value ratio and the inhibition rate (Spearman’s rho = 0.053, 95% confidence interval = − 0.184 to 0.285).

## Discussion

In this study, we demonstrated the seroprevalence of four endemic HCoVs in the pre-pandemic COVID-19 sera of children in the Philippines admitted to hospital with severe respiratory illness and evaluated the SARS-CoV-2 reactivity and neutralization capability of these antibodies. We detected antibodies against all four tested HCoVs in most of 2-month-old individuals. After that, we found a decrease in the frequency of seropositive samples in infants aged between 3 and 11 months old. Over this age, the frequencies increased, starting at 9 to 12 months of age, until a plateau was reached at around 29 months of age. Approximately 22% of the samples showed reactivity against the SARS-CoV-2 spike protein, but hardly any neutralization capability was detected.

Several studies have reported seroprevalence to endemic HCoVs (alpha- and beta-coronaviruses) among children and adults in temperate areas such as the Netherlands, the United States, and China^14–16,21^. These previous studies showed that the presence of these antibodies in sera was age-dependent in adults and in a small number of samples from children. Seroprevalence studies of HCoVs using large numbers of samples from children, especially those under 1 year old, are limited^16^, and there are no reports that analyze the sera of children from tropical areas. To address this gap in the research, we studied more than 150 serum samples from children in the Philippines aged under 1 year old. We detected antibodies against all four HCoVs in most of the 2-month-old individuals, which are likely attributed to acquired maternal antibodies as shown in the previous study^22^ (Fig. 1). We noted a decrease in seropositivity to HCoVs in the samples taken from children aged 3–11 months old due to the disappearance of antibodies; the results suggested that, after this age, individuals start to produce their antibodies against alpha- and beta-coronaviruses, reaching a plateau at 24–29 and 12–17 months of age for alpha- and beta-coronaviruses, respectively. Antibodies against alpha-coronaviruses have been reported to be present in the sera of 50–60% of newborns (0–2 months old) in the United States^14^. The frequencies of seropositive individuals have been found to be lower around 5 months of age, and were reported to increase after that age, until a plateau is reached at 2.5–3.5 years of age^14^. However, other studies have indicated that the plateau of seropositivity to HCoVs is achieved at around 5 years of age^15,16^. Recently, a Canadian study reported that anti-spike antibodies against the four endemic HCoVs were acquired by 10 years of age and were stable thereafter^23^. Our study, like the United States study, identified high levels of seropositivity in newborns and the production of antibodies against the four HCoVs at an earlier age compared to the other studies. Thus, our study demonstrated higher HCoVs seroprevalence at an earlier age in comparison to previous studies conducted in China or Canada. There are some possible reasons for such a difference. First, study site is remote island, and many families are in relatively low socio-economic status^24^. Second, our study subjects were hospitalized children with severe acute respiratory illness. These children might be at a higher risk of getting HCoVs than healthy children. In addition, our result showed that the plateau of seropositivity rate for beta-coronaviruses was reached earlier than for alpha-coronaviruses. Previous studies also indicated higher seropositive rates of beta-coronaviruses in young children than those of alpha-coronaviruses^23,25^. However, seropositive rates may be affected by circulation patterns before sample collection. Further studies are necessary to define the seroprevalence of each virus by analyzing longitudinal samples.

This study revealed that about 20% of children before the COVID-19 pandemic had reactive antibodies to SARS-CoV-2. Previous studies using samples collected before the COVID-19 pandemic revealed varying seroprevalence rates against SARS-CoV-2 using different methodologies; a study in France reported about 6% seropositivity in children^20^, while a study in Finland reported about 80% in children and 40% in adults^25^. The seropositive rate for SARS-CoV-2 depends on the cut-off-value. Some samples in the current study showed the OD value around the cut-off-value (Supplementary Fig. S1). However, the ratio to the cut-off-value for most samples considered positive was substantially higher than the cut-off-value, which indicated some reactivity with the spike protein of SARS-CoV-2. We used a wild strain, a prototype strain detected in Wuhan in 2020 to measure antibodies against SARS-CoV-2. However, SARS-CoV-2 has evolved from wild type to various variants including Alpha, Delta, and Omicron variants. As of January 2023, Omicron sub-variants are predominantly circulating (https://gisaid.org/). Omicron sub-variants such as BQ.1 and XBB are known to have immune escape properties that escape immunity induced by both vaccination and natural infections^26,27^. Our results might differ from the reactivity using recently circulating Omicron sub-variants.

Spike proteins are useful as specific antigens for antibody detection^16^. Regarding the coefficient of determination analysis performed to test the reactivity, interestingly, the highest coefficient of determination with SARS-CoV-2 was found for HCoV-NL63, an alpha-coronavirus, but not for HCoV-HKU1 or HCoV-OC43, which are beta-coronaviruses like SARS-CoV-2 (Fig. 3). The receptors of SARS-CoV-2 and HCoV-NL63 are known to be angiotensin-converting enzyme 2^5,28^. Recently, it was reported that HCoV-NL63 and SARS-CoV-2 share epitopes that are recognized by the humoral response and that the levels of HCoV-NL63 neutralizing antibodies increase after SARS-CoV-2 infection or vaccination^29^. Thus, our results are in line with this report and suggest the highest coefficient of determination between HCoV-NL63 and SARS-CoV-2.

Reactive samples against SARS-CoV-2 showed low neutralization capability in this study (Fig. 4). Our result is in line with previous studies using samples collected before the COVID-19 pandemic^20,30^. Cross-reactivity to SARS-CoV-2 is reported to be due to the recognition of the S2 region, which is more homologous across HCoV spike proteins^19,31^, while the neutralizing capability is achieved by the binding to the receptor-binding domain region of S1 region^32^. Therefore, antibodies that showed reactivity against SARS-CoV-2 in this study may bind to the S2 region. The impact of HCoV and humoral immunity on SARS-CoV-2 has been suggested. SARS-CoV-2 vaccination induces antibodies not only against SARS-CoV-2 but also against HCoVs^33^. In addition, neutralizing monoclonal antibodies against SARS-CoV-2 have been established from B cells derived from HCoVs-antibody-positive individuals^34^. Recently, it was reported that prior HCoV-OC43 S2 region immunity primes neutralizing antibody responses to otherwise sub-immunogenic SARS-CoV-2 spike gene exposure and promotes S2 antibody responses in mice^35^. This study also showed that more than 50% of inhibition was only detected in one sample, but some samples showed 40% inhibition against SARS-CoV-2. These antibodies may increase neutralization capability by SARS-CoV-2 infection. Therefore, some antibodies induced by HCoV infection may have a neutralizing function against various coronaviruses which infect humans. In addition, alpha- and beta-coronaviruses were identified from bats in the Philippines^36^. Although these bat CoVs have not been detected in humans, exposure to unknown CoVs might have produced antibodies that are reactive to SARS-CoV-2.

There are some limitations to the study. Firstly, our study analyzed samples from less than 5 years of children with severe pneumonia. Second, we did not check if these children were infected with HCoVs when samples were collected. Therefore, future studies should include various sample groups, such as heathy children, adults, and elderly population including HCoVs detection method. Third, we measured antibodies against SARS-CoV-2 by using a prototype strain. Our results may not be applicable to currently circulating Omicron sub-variants. To further understand the association between age, and cross-reactive epitopes, future studies should not only be extended to samples from different age groups and the mapping of cross-reactive epitopes.

In conclusion, this study identified the high seropositivity to four endemic HCoVs among children in the Philippines. Infants < 2 months of age had higher rates of seropositivity for four HCoVs, which disappeared after 3 months, likely due to maternal antibodies among young infants. Following its disappearance after 3 months of age, antibodies against HCoVs increased again at 9 to 12 months, probably due to exposures to HCoV. Although 20% of the samples collected from children before the COVID-19 pandemic showed reactivity against the SARS-CoV-2 spike protein, these antibodies had low neutralization capability.

## Methods

### Ethics declarations

The use of clinical samples for the present study was approved by the Research Institute of Tropical Medicine Ethics Review Board in the Philippines (certificate #2008-05-5) and the Tohoku University Ethics Review Board in Japan (certificate #2018-1-69). Written informed consent was obtained from the guardians of all participants.

### Serum samples

A total of 315 serum samples were collected from children in Biliran island, the Philippines, between 2015 and 2018. Participants were aged from 8 days to < 5 years and had presented with severe respiratory illness which were assessed using the Integrated Management of Childhood Illness (IMCI) algorithm by a trained study physician^37^ (Table 1). The samples were inactivated at 56 °C for 30 min. This inactivation process is not expected to have any effect on antibodies^38^. Serum samples from SARS-CoV-2-infected individuals were purchased from commercial laboratory (RayBiotech, GA, USA) and served as positive controls.

**Table 1:** Demographic characteristics of 315 children who were collected before the COVID-19 pandemic (2015–2018) in Biliran, Philippines.

Age (months)	Sex				Total
	Female		Male		
	n	%	n	%	
< 2	17	34.0	33	66.0	50
3–5	13	34.2	25	65.8	38
6–8	13	37.1	22	62.9	35
9–11	12	33.3	24	66.7	36
12–14	12	80.0	3	20.0	15
15–17	8	32.0	17	68.0	25
18–20	9	50.0	9	50.0	18
21–23	7	53.8	6	46.2	13
24–26	9	64.3	5	35.7	14
27–29	9	64.3	5	35.7	14
30–35	7	42.9	10	57.1	17
36–47	14	58.3	10	41.7	24
48–59	9	56.3	7	43.8	16
Total	139	44.1	176	55.9	315
Median (IQR), years	1.29 (0.55–2.3)		0.84 (0.35–1.69)		0.96 (0.42–2.09)

*IQR* interquartile range.

### Detection of antibodies against HCoVs and SARS-CoV-2 spike proteins by ELISA

The ELISA protocol was adapted from a method previously described^25^. In brief, recombinant spike ectodomain proteins from HCoV-229E, HCoV-NL63, HCoV-HKU1, HCoV-OC43, and SARS-CoV-2 were purchased from a commercial laboratory (Sino Biological, Beijing, China) and shown between homology of used each virus in Supplementary Table S1. The proteins were diluted at 1 ng/µL in phosphate buffered saline (PBS) and 100 ng/well was used to coat a 96-well Immulon 1B plate (ThermoFisher Scientific, NY, USA). The plate was then incubated for 30 min at 37 °C. The optimal concentration and dilution of the coating antigens was determined in preliminary assays, prior to serum testing. After washing the unbound antigen (five washes with 300 µL/well of 0.1% Tween-20 in PBS), the plates were blocked with Block Ace (Yukijirushi, Sapporo, Japan) for 1 h at 37 °C. All antibodies and serum samples were diluted in Block Ace. The plates were washed five times between each incubation step. The serum samples were diluted at 1:100, and 100 µL of the dilution was added per well, followed by incubation for 30 min at 37 °C. Positive and negative reference serum samples were used as controls. Horseradish-peroxidase-conjugated goat anti-human IgG secondary antibody (SouthernBiotech, AL, USA), diluted 1:5000, was subsequently added, and plates were incubated for 30 min at 37 °C. The substrate (ELISA POD Substrate Popular, Nacalai tesque, Kyoto, Japan) was added (100 µL/well), and the color reaction was allowed to develop for 10 min in the dark. After incubation, the reaction was stopped with 4N H_2_SO_4_ (50 µL/well), and the optical density (OD) was measured at 450 nm (EPOCH2, BioTek/Agilent Technologies, CA, USA). The background signal from blank wells (non-antigen coated) was subtracted from the signals of all assessed samples. A sample was considered positive if the net OD value was above the cut-off-value, which was calculated for each antigen as the mean net OD + 5 × SD of three negative sera, and was at least 0.1. Antibody level was defined as a ratio to cut-off-value (a ratio of OD value of test sample to the cut-off-value calculated from the mean of the negative control values in each plate).

### SARS-CoV-2 neutralization assay

The VeroE6/TMPRSS2 cell line was obtained from the Japanese Collection of Research Bioresources (JCRB; Osaka, Japan) and cultured in culture medium at 37 °C under a 5% CO_2_. The culture medium, DMEM medium (SIGMA-Aldrich Corp, MO, USA) was supplemented with 1 mg/mL G-418 (FUJIFILM Wako Pure Chemical Corporation, Osaka, Japan), 10% heat-inactivated fetal bovine serum (FBS) (ThermoFisher Scientific, Inc), 100 units/mL penicillin, 100 µg/mL streptomycin, and 25 µg/mL amphotericin B (Nacalai tesque, Inc).

The neutralizing activity of the sera was evaluated in two different assays:

(1) The TCID_50_-based neutralization assay was performed in a similar way as described previously^39^. In brief, the samples were diluted at 1:5 in duplicate with maintenance medium, EMEM medium (SIGMA-Aldrich Corp) containing 2% heat-inactivated FBS, 100 units/mL penicillin, 100 µg/mL streptomycin, and 25 µg/mL amphotericin B. Samples were mixed with an equal volume of medium containing 100 TCID_50_ SARS-CoV-2 (SARS-CoV-2/SMC-VC-1) which isolated from SARS-CoV-2 infected specimens collected on October 29, 2020 in our laboratory, then incubated at 37 °C for 60 min. Then, 96-well tissue culture plates with confluent VeroE6/TMPRSS2 monolayers were infected with the virus/serum mixtures and incubated at 34 °C under a 5% CO_2_. After 7 days, the microplates were observed under an optical microscope for the presence of cytopathic effect (CPE).

(2) For the conventional PRNT, as previously described^40^, VeroE6/TMPRSS2 cells were seeded in 12-well plate with 1 mL of growth medium and incubated until a cell monolayer was formed. The serum samples were diluted in maintenance medium at 1:10 in duplicate, mixed with an equal volume of medium containing 50 plaque forming units of SARS-CoV-2 (SMC-VC-1), and incubated at 37 °C for 60 min. A total of 100 µL of the virus/serum mixture was then added to each well of the monolayer cell cultures in duplicate. The plates were incubated at 37 °C for 60 min. After incubation, 2 mL of overlay medium (avicel and methylcellulose) was added to each well. After 4 days of incubation, the cells were fixed with 4% paraformaldehyde for 60 min at room temperature and stained with 0.25% crystal violet. The number of plaques was counted with the naked eye by two people including one person who was blinded to the sample layout in the 12-well plate. The cut-off-value of inhibition was set at 50%.

All infection experiments with SARS-CoV-2 were performed in a biosafety level 3 (BSL-3) facility at the Tohoku University Graduate School of Medicine, according to local biosafety guidelines and regulations.

### Statistical analyses

All statistical analyses were conducted using GraphPad Prism (San Diego, CA) version 8.3.0. Statistical significance was defined as *P* < 0.05. Mann–Whitney U tests were used to compare differences in the non-parametric observations between the two groups. The Pearson correlation coefficient was used to assess the degree of correlation between antibody levels (ratio to cut-off-value). Spearman’s rank correlation coefficient was used to assess the degree of correlation between antibody levels (ratio to cut-off-value) and percent of inhibition.

## Supplementary Information

Supplementary Information.

## Data Availability

The data sets generated during and/or analyzed during the current study are available from the corresponding author on reasonable request.
