# Supplementary material for: Seroprevalence of four endemic human coronaviruses and, reactivity and neutralization capability against SARS-CoV-2 among children in the Philippines
Source: Sci Rep. 2023 Feb 9;13:2310. doi: 10.1038/s41598-023-29072-3 (PMC9909632; doi:10.1038/s41598-023-29072-3)
Supplement: Supplementary file 1 — Supplementary Information. [file 41598_2023_29072_MOESM1_ESM.docx]

**Supplementary material**

**Title:**

Seroprevalence of four endemic human coronaviruses and, reactivity and neutralization capability against SARS-CoV-2 among children in the Philippines

**Authors:**

Yusuke Sayama^1*^, Michiko Okamoto^1^, Mayuko Saito^1^, Mariko Saito-Obata^1^, Raita Tamaki^2^, Christine Dahlia Joboco^3^, Socorro Lupisan^4^, Hitoshi Oshitani^1^

1. Department of Virology, Tohoku University of Graduate School, Sendai, Japan.
2. Institute of Tropical Medicine, Nagasaki University, Nagasaki, Japan.
3. Biliran Provincial Hospital, Biliran, Philippines.
4. Research Institute for Tropical Medicine, Metro Manila, Philippines.

**Keywords:** endemic human coronaviruses, SARS-CoV-2, antibody, cross-reactivity, neutralization, Philippines, Children

***Corresponding author:** Yusuke Sayama

Department of Virology, Tohoku University Graduate School of Medicine

Address: 2-1 Seiryo-machi, Aoba-ku, Sendai, Miyagi, 980-8575, Japan

Telephone: +81-(0)22-717-8211, FAX: +81-(0)22-717-8212

Email: sayama@med.tohoku.ac.jp


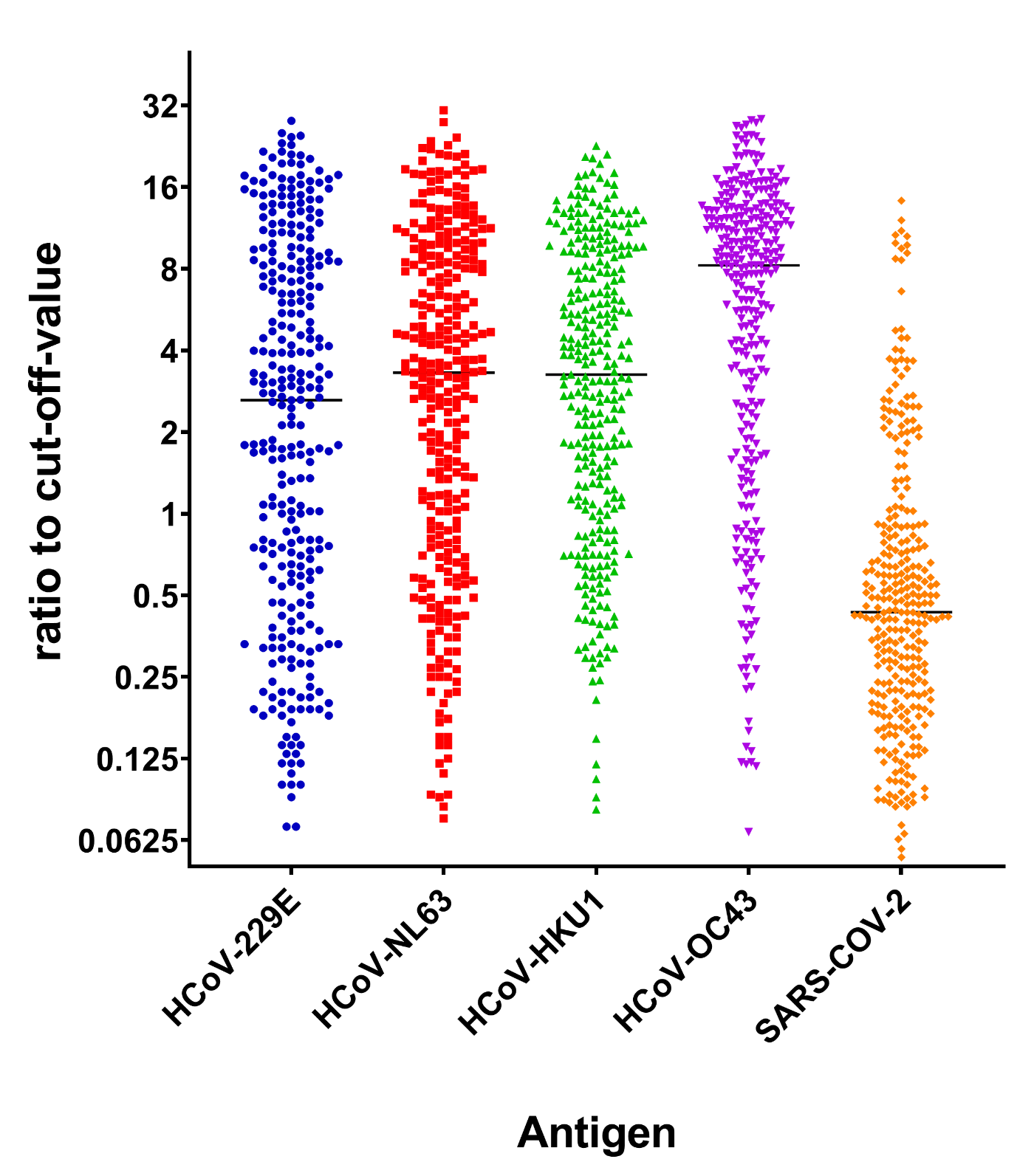
**Supplementary Figure S1.** Serum antibody levels against four HCoVs and SARS-CoV-2 expressed as ratio to cut-off-value. Blue, anti-HCoV-229E; red, anti‑HCoV-NL63; green, anti-HCoV-HKU1; purple, anti-HCoV-OC43; orange, anti-SARS‑CoV-2.

**Supplementary Table S1.** Homology of HCoVs and SARS-CoV-2 spike proteins used in this study.

| Virus | Accession Number | Catalog Number (Sino Biological) | Protein construction | HCoV-NL63 | HCoV-HKU1 | HCoV-OC43 | SARS-CoV-2 |
| --- | --- | --- | --- | --- | --- | --- | --- |
| HCoV-229E | APT69883.1^a^ | 40605-V08B | Cys16-Trp1115 | 53.4% | 19.1% | 18.6% | 17.9% |
| HCoV-NL63 | APF29071.1^a^ | 40604-V08B | Met1-Pro1296 | - | 19.9% | 20.4% | 17.7% |
| HCoV-HKU1 | Q0ZME7.1^b^ | 40606-V08B | Met1-Pro1295 | - | - | 63.4% | 25.2% |
| HCoV-OC43 | AVR40344.1^a^ | 40607-V08B | Met1-Pro1304 | - | - | - | 26.1% |
| SARS-CoV-2 | YP_009724390.1^a^ | 40589-V08B1 | Val16-Pro1213 | - | - | - | - |

a: National Center for Biotechnology Information (NCBI)

b: UniProt (https://www.uniprot.org/uniprotkb/Q0ZME7/entry)

**Supplementary Table S2.** Results of positivity by ELISA

| Age (months) | Number of test samples | HCoV-229E | | | | HCoV-NL63 | | | | HCoV-HKU1 | | | | HCoV-OC43 | | | | SARS-CoV-2 | | | | Four HCoVs and SARS-CoV-2 antigens | | | |
| --- | --- | --- | --- | --- | --- | --- | --- | --- | --- | --- | --- | --- | --- | --- | --- | --- | --- | --- | --- | --- | --- | --- | --- | --- | --- |
|  |  | Negative | % | Positive | % | Negative | % | Positive | % | Negative | % | Positive | % | Negative | % | Positive | % | Negative | % | Positive | % | Negative | % | Positive | % |
| <2 | 50 | 2 | 4.0 | 48 | 96.0 | 5 | 10.0 | 45 | 90.0 | 2 | 4.0 | 48 | 96.0 | 1 | 2.0 | 49 | 98.0 | 39 | 78.0 | 11 | 22.0 | 0 | 0 | 50 | 100 |
| 3-5 | 38 | 12 | 31.6 | 26 | 68.4 | 14 | 36.8 | 24 | 63.2 | 12 | 31.6 | 26 | 68.4 | 7 | 18.4 | 31 | 81.6 | 38 | 100 | 0 | 0 | 4 | 10.5 | 34 | 89.5 |
| 6-8 | 35 | 28 | 80.0 | 7 | 20.0 | 24 | 68.6 | 11 | 31.4 | 17 | 48.6 | 17 | 51.4 | 12 | 34.3 | 23 | 65.7 | 34 | 97.1 | 1 | 2.9 | 8 | 22.9 | 27 | 77.1 |
| 9-11 | 36 | 24 | 66.7 | 12 | 33.3 | 14 | 38.9 | 22 | 61.1 | 21 | 58.3 | 15 | 41.7 | 19 | 52.8 | 17 | 47.2 | 35 | 97.2 | 1 | 2.8 | 8 | 22.2 | 28 | 77.8 |
| 12-14 | 15 | 9 | 60.0 | 6 | 40.0 | 6 | 40.0 | 9 | 60.0 | 4 | 26.7 | 11 | 73.3 | 2 | 13.3 | 13 | 86.7 | 13 | 86.7 | 2 | 13.3 | 0 | 0 | 15 | 100 |
| 15-17 | 25 | 12 | 48.0 | 13 | 52.0 | 8 | 32.0 | 17 | 68.0 | 3 | 12.0 | 22 | 88.0 | 4 | 16.0 | 21 | 84.0 | 19 | 76.0 | 6 | 24.0 | 2 | 8.0 | 23 | 92.0 |
| 18-20 | 18 | 6 | 33.3 | 12 | 66.7 | 6 | 33.3 | 12 | 66.7 | 6 | 33.3 | 12 | 66.7 | 3 | 16.7 | 15 | 83.3 | 11 | 61.1 | 7 | 38.9 | 1 | 5.6 | 17 | 94.4 |
| 21-23 | 13 | 5 | 38.5 | 8 | 61.5 | 6 | 46.2 | 7 | 53.8 | 1 | 7.7 | 12 | 92.3 | 1 | 7.7 | 12 | 92.3 | 11 | 84.6 | 2 | 15.4 | 0 | 0 | 13 | 100 |
| 24-26 | 14 | 4 | 28.6 | 10 | 71.4 | 2 | 14.3 | 12 | 85.7 | 2 | 14.3 | 12 | 85.7 | 2 | 14.3 | 12 | 85.7 | 9 | 64.3 | 5 | 35.7 | 1 | 7.1 | 13 | 92.9 |
| 27-29 | 14 | 2 | 14.3 | 12 | 85.7 | 2 | 14.3 | 12 | 85.7 | 2 | 14.3 | 12 | 85.7 | 0 | 0 | 14 | 100 | 5 | 35.7 | 9 | 64.3 | 0 | 0 | 14 | 100 |
| 30-35 | 17 | 5 | 29.4 | 12 | 70.6 | 3 | 17.6 | 14 | 82.4 | 2 | 11.8 | 15 | 88.2 | 1 | 5.9 | 16 | 94.1 | 11 | 64.7 | 6 | 35.3 | 1 | 5.9 | 16 | 94.1 |
| 36-47 | 24 | 4 | 16.7 | 20 | 83.3 | 0 | 0 | 24 | 100 | 1 | 4.2 | 23 | 95.8 | 0 | 0 | 24 | 100 | 13 | 54.2 | 11 | 45.8 | 0 | 0 | 24 | 100 |
| 48-59 | 16 | 1 | 6.3 | 15 | 93.8 | 0 | 0 | 16 | 100 | 0 | 0 | 16 | 100 | 0 | 0 | 16 | 100 | 8 | 50.0 | 8 | 50.0 | 0 | 0 | 16 | 100 |
| Total | 315 | 114 | 36.2 | 201 | 63.8 | 90 | 28.6 | 225 | 71.4 | 73 | 23.2 | 241 | 76.5 | 52 | 16.5 | 263 | 83.5 | 246 | 78.1 | 69 | 21.9 | 25 | 7.9 | 290 | 92.1 |
